# Impact of cumulative family risk on Chinese primary school children’s flourishing: parallel mediating roles of teacher–student and peer relationships

**DOI:** 10.3389/fpsyg.2026.1922081

**Published:** 2026-10-26

**Authors:** Liu Bin, Yan Chenghai, Liu Yinbo

**Affiliations:** 1 School of Humanities and Social Science, Xi’an Jiaotong University, Xi’an, Shaanxi, China; 2 Xi’an University, Xi’an, Shaanxi, China; 3 Shandong University of Political Science and Law, Jinan, Shandong, China

**Keywords:** cumulative family risk, flourishing, peer relationship, primary school children, teacher–student relationship

## Abstract

**Introduction:**

Children’s flourishing has become a central outcome in the study of child development, but its link with the cumulative burden of family risk remains poorly understood. This study examined whether two school-based relational resources—relationships with teachers and with peers—account for the association between cumulative family risk and flourishing in primary school children.

**Methods:**

A questionnaire survey was completed by 1,673 fourth and fifth-grade children in Shaanxi province, Northwest China. A parallel mediation model with bootstrap confidence intervals was estimated, with sex, only-child status, and household registration entered as covariates.

**Results:**

Cumulative family risk was negatively associated with flourishing, and the decrement was approximately linear across the number of risk factors rather than accelerating at higher levels. Teacher–student and peer relationships each carried an independent part of this association, and the peer pathway carried the larger share.

**Discussion:**

Interventions should give priority to children exposed to several family risks. Where school resources are limited, restoring peer relationships deserves precedence, while the teacher–student relationship retains an independent contribution and should not be neglected.

## 1 Introduction

In contemporary positive psychology, mental health is understood less as the absence of disease than as the realization of individual potential (Keyes, 1998; Huppert and So, 2013). Within this paradigm, the core construct of flourishing is regarded as an individual’s high-level and balanced state that includes emotional well-being, developed psychological functions, and integration within social systems (Rule et al., 2024; Keyes, 1998; Tang et al., 2016; Huppert and So, 2013). In primary school children, flourishing reflects the capacity to sustain well-being while coping with environmental pressure. An individual’s sense of happiness is evaluated by their emotional balance and life satisfaction (Pan et al., 2025; Huppert and So, 2013). A dual-factor formulation reaches a similar conclusion: family, peer, and teacher–school relationships jointly distinguish positive mental health profiles from other groups (Rose et al., 2025), which supports treating flourishing as a holistic well-being outcome rather than merely the absence of distress.

The Ecological Systems Theory posits the family as the most persistent and pervasive micro-system during an individual’s development (Bronfenbrenner and Morris, 1998; Sirisety et al., 2026). Thus, the quality of the family environment is regarded as shaping whether a child’s psychological resources are “abundant” or “depleted.” Closeness and cohesion within the family are among the most reliable correlates of an individual’s subjective well-being (Gu et al., 2025). However, the family can also be a setting in which risk factors accumulate. The family’s failure to provide the necessary resource support, along with the presence of systematic stressors, has been linked to lower levels of children’s flourishing. Most prior studies focused on single isolated family risk factors, while the cumulative and synergistic associations between multiple co-occurring family risks and children’s flourishing remain underexplored.

However, few studies have clarified how family risks are related to children’s positive development through the interpersonal resources of the school microsystem, and in particular whether the two core school relationships—teacher–student and peer relationships—carry this association to a comparable degree. To address these gaps, this study aims to examine the negative association between family cumulative risk and primary school children’s flourishing, and further to examine the independent and relative mediating roles of teacher–student relationship and peer relationship. The two channels are therefore compared directly rather than treated as equivalent. To clarify the cross-system logic of family adversity, this study integrates two classic developmental theories as the theoretical basis of the research model. The Family Stress Model accounts for within-family risk accumulation, while Ecological Systems Theory accounts for cross-system risk transmission from the family microsystem to the school microsystem, jointly supporting the parallel mediation hypothesis. Because the data are cross-sectional, the mediation model is interpreted throughout as a theory-driven decomposition of covariance rather than as a causal chain.

## 2 Literature review

### 2.1 Impact of accumulated family risks on flourishing

Ecological Systems Theory posits that for children’s development, the family microsystem offers the most direct environment. The prerequisites for an individual to reach flourishing within this system are abundant resources and the quality of interactions (Bronfenbrenner, 1979; Lu, 2023). A systematic review of previous studies on family adversity shows that family risks are often viewed from three dimensions: family structural vulnerability (e.g., parental divorce, experiences of left-behind, living without parents), family resources and socioeconomic factors (e.g., lack of education, unemployment, and poverty), and family interactions (e.g., challenges posed by modern technology) (Zhao and Wang, 2025; Liu et al., 2026).

Family structure and socioeconomic status (SES), including parents’ education and income are generally regarded as the material foundations for children’s development. The social causation hypothesis states that children from high SES families possess abundant resources for self-development (Wadsworth and Achenbach, 2005). Although the psychological environment has a direct impact, the family’s physical environment and SES are key carriers of the social causation hypothesis. These set the initial boundaries within which children can access developmental resources (Chen et al., 2025). Parents’ educational level not only transmits cultural capital but also influences the complexity of family cognition and compensation strategies for stress, while family income directly affects parents’ emotional states and parenting quality via resource stability (Wang and Jia, 2023). Meanwhile, family structure stability, such as parents’ marital status is widely recognized as a reliable predictor of internalizing problems in children, such as depression and reduced happiness (Chen et al., 2024). Therefore, when assessing cumulative risk, including structural and resource factors more objectively replicates their developmental background (Wu et al., 2023).

Family atmosphere and parenting behaviors are mid-level hubs for risk transmission. Persistent conflicts and a disharmonious family environment can have a negative external impact, directly reducing children’s psychological security, and hinder their thriving (Lu et al., 2024; Ran et al., 2021). Moreover, parental marital conflict can significantly undermine children’s subjective well-being by reducing their satisfaction with basic psychological needs (He et al., 2023). While assessing the interaction dimension within the framework of attachment theory, parental rejection, which typically represents negative parenting styles exhibited high explanatory power (Chen et al., 2025; Gu et al., 2025). Numerous empirical studies indicate that parental rejection not only directly impacts children’s tendencies towards depression but also reduces the necessary internal buffering against external pressures by undermining their core self-evaluation (Wang and Jia, 2023; Yu et al., 2024). Furthermore, parental phubbing—parents’ absorption in their smartphones during interactions with their children—has been linked to higher depressive symptoms and psychological distress in children and adolescents (Li et al., 2025; Xie and Xie, 2020). Phubbing is therefore not an isolated lapse of attention but a recurring feature of the family environment, one that repeatedly interrupts parents’ responsive engagement with the child (Li et al., 2025). Parents’ excessive reliance on smartphones while accompanying their children essentially deprives them of the opportunities to receive synchronous social support, directly inhibiting their learning engagement and the development of positive emotions (Hu et al., 2023; Yuan et al., 2021). Currently, researchers are increasingly calling for assessing the current risks to family systems, noting that ignoring digital risk factors may pose challenges and compromise the ecological validity of the assessment (Garrido et al., 2024; Chotpitayasunondh and Douglas, 2016; Xie and Xie, 2020). Unlike traditional family risks, parental phubbing is a prevalent digital interaction risk unique to contemporary families, so it is incorporated to improve the ecological validity of the cumulative risk model. Therefore, incorporating such factors into risk assessment systems is necessary for modifying the traditional risk model.

When examining the influence of family environmental variables on children’s development, the academic research paradigm has gradually shifted from the earlier “Single Risk Model” to the “Cumulative Risk Model”. The single-risk perspective tends to explore the predictive role of specific variables (such as poverty or divorce); however, in real-life situations, family risks often tend to co-occur. An individual’s compensatory ability for a single risk factor usually falls within the threshold of psychological resilience, while the superposition of multiple risk factors in both time and space dimensions causes impairment in an individual’s adaptive function (Evans and Marcynyszyn, 2004). The rationale for a cumulative index rests on two observations. First, risks co-occur: individuals who bear a risk in one domain are likelier to face risks in another (Evans and Marcynyszyn, 2004). In a real social ecosystem, low SES often indicates higher frequency of parental conflict, often triggering parents’ adoption of negative parenting styles through the cascading effect of the Family Stress Model (Chen and Wu, 2025). Second, cumulative risk is more than the simple sum of individual factors; the accumulation itself carries an added toll. When the total number of risk factors exceeds the maximum capacity of a child’s psychological defense system, the continuous deprivation of basic psychological needs causes severe systemic damage (Liu et al., 2026). Because the conventional cumulative risk index is additive by construction, it cannot by itself detect interactions among risk factors; whether the decrement associated with each additional risk grows disproportionately must therefore be tested explicitly (Evans et al., 2013). Scholars have put forward two competing hypotheses regarding the correlation between cumulative family risk and children’s positive development: the linear additive model and the nonlinear threshold model. The current study incorporates the quadratic term of CFR into regression analysis to compare which pattern fits the sample data. Therefore, assessing cumulative risk is more reliable for predicting the degree of loss in children’s sense of flourishing than tracking the individual characteristics of each risk factor (Liu et al., 2025). A person-centered complement to the variable-centered index used here is also available: latent class analyses have identified distinct configurations of poverty-related family risk that predict divergent longitudinal outcomes for parents and children (Cooper et al., 2025), supporting the treatment of family risk as a multidimensional construct rather than as a single severity continuum. Hence, we propose the following hypothesis:

*H1*: CFR has a significant negative predictive effect on primary school children’s flourishing, and this effect follows a cumulative linear pattern, such that each additional risk factor is associated with a further decrement in flourishing. The quadratic term of CFR is tested additionally to examine whether the decrement accelerates at higher levels of risk, as implied by the nonlinear threshold model.

### 2.2 The mediating role of teacher–student and peer relationships

Individual development is shaped by the interactions between microsystems, such as family and school. When the family microsystem becomes dysfunctional, negative psychological experiences are presumed to spill over across system boundaries, accompanying poorer interpersonal adaptation at school. This is manifested in two core relational domains: the teacher–student relationship and peer relationships, within which risk accumulates (Lu et al., 2024; Jia et al., 2025; Zhao and Wang, 2025).

First, CFR had a negative predictive effect on the teacher–student relationship. According to the family stress model, Chen and Wu et al. (2025) showed that family financial difficulties and long-term care needs may trigger parental conflicts and deteriorate the parent–child relationship, thus producing negative spill-over effects. Such systematic family pressure weakens social and emotional abilities, leading to detachment or conflict in the teacher–student interaction (Jia et al., 2025). These relationships are shaped by teachers’ responses to students’ psychological needs, as well as balancing between emotional support and the classroom structure (Bear, 2020). At the family level, negative risks tend to undermine an individual’s interpersonal integration into the school environment (Wu et al., 2025; Lu, 2023). Poor parent–child relationships such as, psychological or physical abuse and neglect can exacerbate insecurity and avoidance of intimacy, thus reducing their social interaction skills (Li et al., 2023). In the digital age, parental phubbing reduces children’s psychological security at school, thereby reducing their motivation to establish positive teacher connections (Hu et al., 2023). A bidirectional relationship between family dysfunction and an individual’s impaired psychological flexibility further exacerbates social withdrawal or adaptation disorders among children during interactions with teachers (Li et al., 2025).

Second, the teacher–student relationship predicts children’s sense of flourishing. It is associated with higher subjective well-being and positively predicts academic engagement (Brieba-Fuenzalida, 2025). Meta-analytic evidence further indicates that affective teacher–student relationships are associated with students’ school engagement and achievement, with medium-to-large associations for engagement (Roorda et al., 2011), and that teacher–student relationship quality is implicated in students’ adjustment across school transitions (Longobardi et al., 2019). Longitudinal work also indicates that parent–child and teacher–student relationships jointly predict later adjustment through the mediating role of self-esteem (Jia et al., 2024). An effective teacher–student relationship can alleviate and buffer the family’s negative impact. A previous study of left-behind children found that teacher support mediated the association between CFR and social adaptation by enhancing self-worth (Zhang et al., 2025). Thus, as family-related risks accumulate, a poorer teacher–student relationship would imply the loss of external compensatory resources and a lower standing on the “positive psychological function” dimension of flourishing (Rule et al., 2024). For school connection, the core manifestation is the quality of the teacher–student relationship, which mitigates the association between family risks and depression. A decline in the quality of school connections implies that the risks to individuals remain unmitigated (Xia et al., 2025). Conversely, teacher–student conflict is associated with greater risk related to a child’s standing in the classroom, including involvement in bullying behaviors and exposure to peer victimization (Marengo et al., 2021). Accordingly, we expect that part of the association between CFR and children’s flourishing is accounted for by individual differences in the quality of the teacher–student relationship. Therefore, we postulate the following hypothesis:

*H2*: The teacher–student relationship plays a significant mediating role between CFR and primary school children’s flourishing.

Beyond family, peer friendships provide security and support for children’s development (Havewala et al., 2019; Zhang et al., 2021). Peer relationships have been examined in relation to CFR and serve as a key indicator for measuring social well-being and interpersonal integration (Yu et al., 2024). As family risk factors accumulate, children report less social support and fewer interpersonal resources at school. Effective family functioning is significantly and positively correlated with high-quality friendships (Kim, 2021; Kaufman et al., 2020). The family environment shapes a child’s character and social skills, laying the foundation for the establishment of peer relationships within the school setting (Yang et al., 2025). Negative parenting, such as material punishment, is associated with lower subjective well-being and with a reduced capacity to benefit from friendships (Zhou et al., 2025a, 2025b; Guo et al., 2020). Parental conflict has been linked to lower satisfaction of the child’s basic psychological needs. Consequently, children may exhibit higher hostility or withdrawal behaviors in their peer interactions (He et al., 2023). Peer and teacher support have been examined as resilience factors on this pathway: in a study linking adverse childhood experiences to adolescent affective distress through basic psychological needs, both sources of support were tested for their buffering role (Zhou et al., 2025a, 2025b). A large sample study found that family economic pressures are associated with discrimination, which in turn is associated with a more isolated position within the peer group (Wu et al., 2023).

However, high-quality peer relationships can significantly promote an individual’s flourishing. Zeng et al.’s (2025) longitudinal study found that peer relationships could continuously predict an individual’s psychological qualities through self-esteem’s mediating effect. Children’s high-quality friendships, can, to some extent, buffer the psychological harm caused by family dysfunction. High friendship quality significantly alleviated the direct negative impact of material punishment on sleep quality and happiness (Guo et al., 2020). In a longitudinal study of primary school children, friendship quality mediated the association between family functioning and depressive symptoms while teacher–student relationship acted as a moderator, suggesting that the two relational resources operate as distinguishable rather than interchangeable channels (Yang et al., 2025). In contrast, if CFR undermines peer relationships, resulting in bullying or social isolation, children encounter a crisis in their sense of belonging. Bullying directly leads to the collapse of school belongingness, which in turn, worsens teacher–student relationships (Li et al., 2026). An absence of a supportive peer system increases individuals’ sense of moral evasion, leading to a loss of social functioning required to thrive in society (Bao et al., 2025).

*H3*: Peer relationships play a significant mediating role in the relationship between CFR and children’s flourishing.

### 2.3 Teacher–student and peer relationships as parallel mediators

The two school relational systems are closely interwoven rather than independent. Teacher–student and peer relationships are moderately correlated in childhood, and each is associated with the other over time (Hughes and Chen, 2011; Jia et al., 2025). However, the direction of that prospective influence is not settled, and the available evidence points in both ways depending on the age of the sample. In a three-wave study of students followed across Grades 2 to 4, teacher–student relationship quality predicted subsequent peer academic reputation, whereas peer academic reputation did not predict teacher–student relationship quality (Hughes and Chen, 2011). By contrast, in a three-wave study of 586 children followed annually from Grades 4 to 6—that is, the same age range as the present sample—sociometric popularity predicted later teacher–child support, which in turn predicted later popularity, indicating that the peer system can initiate the transactional sequence (De Laet et al., 2014). Evidence from a large Chinese sample is consistent with the latter pattern: in a two-wave study of 2,776 fourth graders reassessed in Grade 6, peer acceptance and peer rejection measured by peer nomination significantly predicted teacher–student relationship 2 years later (Deng et al., 2025). Cross-lagged work further shows that being bullied erodes school belonging, which subsequently worsens teacher–student relationships (Li et al., 2026), and that peer characteristics jointly predict teacher–student conflict and closeness (Hernández et al., 2021). Taken together, the relation between the two relational systems is best described as transactional and age-graded; no consistent temporal priority of one over the other can be assumed *a priori*.

A second consideration argues against imposing an ordering. The mechanism by which teachers shape a child’s standing among classmates—the teacher as a social referent—is real but demonstrably path-limited. A meta-analysis of 297 studies and 1,475 effect sizes found that it was predominantly the negative aspects of the teacher–student relationship that predicted peer relationships, and that teacher–student relationship quality partially mediated the association between student behavior and peer relationships (Endedijk et al., 2022). Consistent with this, a longitudinal study of 1,420 fifth graders with observed teacher behavior found that teachers functioned as social referents for peer status only through their negative behavior: negative teacher behavior predicted peers’ perceptions that the teacher disliked the target child, which in turn predicted peers’ disliking of that child, whereas the corresponding positive pathway was not supported (Hendrickx et al., 2017). Because the present study assesses the positive quality of both teacher–student and peer relationships, the teacher-to-peer mechanism would be expected to operate at its weaker end in this age group. Imposing a teacher-to-peer ordering would therefore build the model on the less well-supported half of the available evidence.

For these reasons, the two relational systems were specified as parallel, rather than serial, mediators. In a multiple mediator model, each mediator is entered simultaneously and each specific indirect effect is estimated net of the other, which yields the unique contribution of each channel and permits a formal contrast of their magnitudes (Preacher and Hayes, 2008). A parallel specification does not assume that the two mediators are unrelated; their association is retained and is controlled by their mutual adjustment in the same equation. What is given up is only the claim about temporal precedence, which cross-sectional data cannot adjudicate. This specification also makes a question answerable that a serial model cannot: not which relationship comes first, but how much of the family-risk transmission each relationship carries. The question is substantively meaningful, because student–student and student–teacher relationships have been shown to carry differential implications for later adjustment (Kim, 2021). Recent person-centered evidence is consistent with this treatment: in fourth to sixth-graders, profiles that combine peer and teacher–student relationships predict early adolescents’ mental health, indicating that the two relational systems form configurations that matter jointly rather than substituting for one another (Demol et al., 2025).

We therefore hypothesize that both relationships mediate the association between CFR and flourishing, and that they do so unequally. Regarding the direction of the asymmetry, three considerations lead us to expect a larger indirect effect through peer relationships. First, by middle childhood the peer group has become a consolidated social context in which children spend the largest share of their school day, and peers increasingly serve as the primary source of daily social validation (Rubin et al., 2006). Second, as noted above, the teacher’s leverage over peer standing is concentrated in negative behavior and is weaker for positive relationship quality, which is what the present study measures (Endedijk et al., 2022; Hendrickx et al., 2017). Third, the longitudinal evidence obtained in the same grade range as the present sample points from peer relationships to teacher–student relationships rather than the reverse (De Laet et al., 2014; Deng et al., 2025).

*H4*: Teacher–student relationships and peer relationships each independently mediate the association between CFR and primary school children’s flourishing.

*H5*: The specific indirect effect through peer relationships is significantly larger than the specific indirect effect through teacher–student relationships.

## 3 Materials and methods

### 3.1 Participants and procedure

We originally recruited 1750 participants aged 10–11 years from primary schools in Shaanxi province, Northwest China. A total of 10 primary schools were involved. For each school, two grades, fourth and fifth grades, were selected. Two classes were randomly sampled from each grade. The final result was 4 classes per school, with a total of 40 classes as the sample. Due to missing data (withdrawal, leave of absence, transfer to another school), 77 cases were removed by listwise deletion, leaving a final valid sample of 1,673 individuals (95.6% of those recruited), with 48.06% of female participants. The sample comprised 826 fourth graders (49.37%) and 847 fifth graders (50.63%). With respect to the covariates used in this study, 40.65% of the children were only children, and 24.51% held rural household registration.

Data were collected using paper-and-pencil questionnaires administered in group sessions at school. Participation was anonymous and voluntary. The study was approved by the Ethics Committee of Xi’an University and was conducted in accordance with the ethical standards of the institutional review board and with the 1964 Declaration of Helsinki and its later amendments. Written permission was obtained from the participating schools; parents or legal guardians were informed of the study through the schools and were given the opportunity to decline their child’s participation; oral assent was obtained from each child before the questionnaires were administered.

### 3.2 Measurements

#### 3.2.1 The flourishing scale (FS)

Flourishing was measured using the Chinese version of the flourishing scale (FS) (Tang et al., 2016). The FS has eight self-assessment items and uses a 7-point Likert scale for measurement (1 = strongly disagree, 7 = strongly agree). The higher the score, the more positive psychological resources and social functions an individual has. This scale demonstrated high reliability in a sample of Chinese students (Liu et al., 2020), with a Cronbach’s alpha coefficient of 0.912 in this study. Confirmatory factor analysis supported the unidimensional structure of the scale in the present sample: *χ*^2^(20) = 181.37, *p* < 0.001; CFI = 0.978, TLI = 0.970, RMSEA = 0.069 [90% CI (0.060, 0.079)], SRMR = 0.024. Because the chi-square test is sensitive to sample size, model fit was evaluated primarily with the relative fit indices, all of which met conventional criteria. Standardized factor loadings ranged from 0.637 to 0.806 (all *p* < 0.001), composite reliability was 0.914, and average variance extracted was 0.570, indicating adequate convergent validity.

#### 3.2.2 Teacher–student relationship scale (TSR)

To examine the perceived state of the teacher–student relationship among students, we used Li et al. (2010) “teacher–student relationship” sub-questionnaire of the “school environment questionnaire” (Li et al., 2010). It consists of five items, such as “When I’m with my teacher, I feel very happy.” This scale adopts a 5-point scoring system, and the participants’ responses are measured by the degree of agreement with each statement. A higher score represents students’ perception of a more positive teacher–student relationship. The scale’s Cronbach’s alpha was 0.861.

#### 3.2.3 Peer relationship scale (PRS)

To examine the perceived status of peer interaction relationships among students, we used Li et al.’s (2010) “peer relationship” sub-questionnaire of the “school environment questionnaire” (Li et al., 2010). It consists of five items, such as “I seriously consider all the opinions my good friends give me.” The participants’ responses are measured by the degree of agreement with each statement on a 5-point scoring system. A higher PRS score indicates students’ perception of more positive peer relationships. The scale’s Cronbach’s alpha coefficient was 0.843.

#### 3.2.4 Cumulative family risk (CFR)

In previous cumulative risk studies (Bao et al., 2014; Gerard and Buehler, 2004; Gan et al., 2022; Zhao and Wang, 2025), some risk factors were continuous variables, while others are assessed using single questions. CFR is primarily divided into three categories: family structure risk, family resource shortage risk, and family atmosphere risk. Family structure risk is measured by family structure; family resource shortage risk is evaluated using parents’ educational level and financial difficulties; and family atmosphere risk is assessed through family intimacy, parental rejection, and parental phubbing.

1) *Family structure*: To measure participants’ family structure, a single item, “Which family members are currently living with you?” was used (Dong and Lin, 2011). Neither the “biological father” nor the “biological mother” living with the child was coded 1 (risk), while the rest were coded as 0 (no risk).
2) *Parental educational level*: To measure parents’ educational attainment, two separate items were used (Bao et al., 2014). Both parents’ educational level below the “high school” level was coded 1 (risk), otherwise 0 (no risk).
3) *Family financial hardship*: The Family Economic Stress Scale measured family financial hardship, employing Wadsworth and Compas’s (2002) instrument, which was subsequently revised by Wang et al. (2010). This scale consists of four questions (e.g., “My family doesn’t have enough money to buy a good house”) scored on a 5-point Likert scale. In the cumulative risk study, scores ≥ the 75th percentile were coded 1, the rest as 0. The scale’s Cronbach’s α coefficient was 0.858 in this study.
4) *Family atmosphere*: The Family Intimacy and Adaptability Scale, developed by Olson in 1982 and included in the Psychological Health Assessment Scale, measured family atmosphere as used in a previous study (Barnes et al., 1982). This scale consists of 16 questions scored on a 5-point Likert scale. Higher scores indicate greater family intimacy. Because higher scores denote lower risk on this indicator, scores ≤ the 25th percentile (i.e., low intimacy) were coded 1 and the rest as 0; 24.4% of the sample was classified as being at risk. The Cronbach’s α coefficient was 0.900 in this study.
5) *Parent rejection*: The Chinese version of the simplified parenting style questionnaire (S-EMBU-C) was used to measure the parent rejection dimension of the parent–child relationship. This questionnaire consists of 11 questions scored on a 4-point scale. A higher score reflects a worse parent–child relationship. Consistent with the direction of the construct, scores ≥ the 75th percentile (i.e., high parental rejection) were coded 1 and the rest as 0; 25.8% of the sample was classified as being at risk. The Cronbach’s α coefficient was 0.909 in this study.
6) *Parental phubbing*: The Parent Phubbing Scale was used to measure parental phubbing (Zu et al., 2022). This scale consists of nine items and uses a Likert 5-point scoring method. A higher score reflects more severe parental phubbing behavior. Scores ≥ the 75th percentile were coded as 1 and the rest as 0; 28.8% of the sample was classified as being at risk. Reverse-worded items were recoded before scoring so that a higher score consistently indicated more severe phubbing. The scale’s Cronbach’s α coefficient was 0.831 in this study.

Drawing on Bronfenbrenner’s Ecological Systems Theory and Evans and Marcynyszyn’s (2004) cumulative risk framework, six family risk indicators covering structural, socioeconomic, and parent–child interaction domains were selected. These well-validated measures include the emerging digital risk of parental phubbing to improve ecological validity. Consistent with mainstream practices, unweighted binary summation was utilized. No universal weighting standard exists across existing studies, and artificial weights would bring subjective bias. The cumulative risk theory centers on the count of concurrent adversities rather than single-risk severity; each risk equally drains children’s psychological resources. All continuous indicators were dichotomized via percentile thresholds and were thresholded in the direction consistent with the construct, such that a code of 1 denoted greater risk on every indicator; for the indicator on which higher scores denote better functioning (family intimacy), the threshold was applied at the lower tail. The total CFR score (0–6) was summed from the six binary items, with higher scores indicating more family risks. In the present sample CFR ranged from 0 to 6, with M = 1.672 and SD = 1.43. Table 1 reports the descriptive statistics of the six indicators.

**Table 1:** Descriptive statistics of the six cumulative family risk indicators.

Indicator	Coded 1 when	% at risk	M	SD	α
1. Family structure	not living with both biological parents	15.8%	0.158	0.365	—
2. Parental education	both parents below high school	52.3%	0.523	0.500	—
3. Financial hardship	≥ 75th percentile	20.1%	0.201	0.401	0.858
4. Family intimacy	≤ 25th percentile (low intimacy)	24.4%	0.244	0.430	0.900
5. Parental rejection	≥ 75th percentile (high rejection)	25.8%	0.258	0.438	0.909
6. Parental phubbing	≥ 75th percentile	28.8%	0.288	0.453	0.831

### 3.3 Statistical analysis

SPSS 25.0 and Stata 16.0 were used for data analysis. The Harman single-factor test was utilized to examine common method bias, and Pearson’s correlation analysis was performed to examine the correlations among the study variables. To test H1, the linear and quadratic terms of CFR were entered simultaneously in a regression on flourishing. A significant quadratic term would indicate that the decrement associated with each additional risk accelerates or saturates at higher levels of risk, whereas a non-significant quadratic term is consistent with the linear additive pattern specified in H1. This regression was estimated with 95% bias-corrected bootstrap confidence intervals based on 5,000 resamples.

The hypothesized mediation model was tested using Hayes (2022) PROCESS v4.1 Model 4, which specifies teacher–student relationship and peer relationship as parallel mediators. In this specification both mediators are entered simultaneously, so each specific indirect effect is estimated net of the other and reflects the unique contribution of that channel; the two specific indirect effects can therefore be formally contrasted (Preacher and Hayes, 2008). Sex (1 = male, 0 = female), only-child status (1 = only child, 0 = not), and household registration (0 = rural, 1 = not rural) were entered as covariates in all equations. Bootstrapping based on 5,000 resamples was used to obtain 95% percentile bootstrap confidence intervals for the total, direct, and indirect effects, and a pairwise contrast of the two specific indirect effects was requested to test H5.

Two aspects of model specification were examined, both estimated with sex, only-child status, and household registration entered as covariates and 5,000-resample bias-corrected bootstrap confidence intervals. First, the products of CFR with each mediator (constructed from grand-mean centered variables) were tested to examine whether the association between each mediator and flourishing varies across levels of family risk; the size and significance of these products determine whether the additive specification used here is tenable. Second, because the ordering of two mediators is not identified in a cross-sectional design, both possible serial orderings (CFR → TSR → PRS → FS, and CFR → PRS → TSR → FS) were additionally estimated as sensitivity analyses. All three specifications share the same final equation and are saturated with respect to the observed covariance matrix, so they necessarily yield an identical total indirect effect; the sensitivity analysis therefore documents what the data cannot adjudicate rather than testing a hypothesis.

### 3.4 Common method Bias

Since all data in this study were obtained from participants’ self-reports, common method bias may exist. Although we adopted anonymous responding to control for this issue, to further enhance the rigor of the study, we conducted the Harman’s one-factor test to examine common method bias. We performed an exploratory factor analysis (EFA) on the variables of cumulative family risk, teacher–student relationship, peer relationship, and flourishing. The explanatory power of the first common factor was examined in an unrotated principal component analysis. The results showed that there were 12 factors with eigenvalues greater than 1, which collectively accounted for 63.450% of the total variance. The first factor explained 24.443% of the variance, which was below the 40% cutoff criterion.

## 4 Results

### 4.1 Preliminary analyses

Table 2 presents the means, standard deviations, and zero-order correlations among the study variables. As expected, CFR was negatively correlated with FS (*r* = −0.413, *p* < 0.001), TSR (*r* = −0.329, *p* < 0.001), and PRS (*r* = −0.406, *p* < 0.001). Additionally, FS was positively correlated with TSR (*r* = 0.470, *p* < 0.001) and PRS (*r* = 0.566, *p* < 0.001), and TSR and PRS were moderately and positively correlated with each other (*r* = 0.508, *p* < 0.001). These zero-order correlations correspond closely to the corresponding paths of the covariate-adjusted model reported below (a₁ = −0.332, a₂ = −0.410, c = −0.416), which confirms that Table 1 and the mediation model were estimated on the same re-coded data. The distribution of the CFR index was as follows: 0 risks, 23.1% (*n* = 387); 1 risk, 30.5% (*n* = 510); 2 risks, 20.7% (*n* = 347); 3 risks, 12.5% (*n* = 209); 4 risks, 8.7% (*n* = 145); 5 risks, 3.8% (*n* = 64); and 6 risks, 0.7% (*n* = 11).

**Table 2:** Means, standard deviations, and zero-order correlations among the study variables (*N* = 1,673).

Variable	M	SD	1	2	3	4
1. CFR (0–6)	1.672	1.43	—			
2. TSR (1–5)	3.055	1.046	−0.329***	—		
3. PRS (1–5)	4.106	0.858	−0.406***	0.508***	—	
4. FS (1–7)	5.714	1.240	−0.413***	0.470***	0.566***	—

*N* = 1,673. CFR = cumulative family risk; TSR = teacher–student relationship; PRS = peer relationship; FS = flourishing. ***p < 0.001.

### 4.2 Linear and quadratic effects of cumulative family risk

CFR was significantly and negatively associated with children’s flourishing, *β* = −0.416, SE = 0.022, 95% CI [−0.460, −0.372], *p* < 0.001, after adjustment for sex, only-child status, and household registration; the corresponding zero-order correlation appears in Table 2 (*r* = −0.413). This supports H1. When the quadratic term was entered alongside the linear term, it was not significant, *β* = −0.122, *B* = −0.023, BootSE = 0.012, 95% BootCI [−0.046, 0.001], *p* = 0.056. The confidence interval of the quadratic term included zero, indicating that within the observed range of risk the decrement in flourishing was additive rather than accelerating or saturating. No threshold beyond which functioning declines disproportionately could be identified in this age group.

### 4.3 Parallel mediation analysis

Table 3 presents the regression results of the parallel mediation model, and Figure 1 displays the path model. All equations were estimated with sex, only-child status, and household registration entered as covariates. Both unstandardized (B) and completely standardized (β) coefficients are reported; because the focal variables retained their original metrics, the two sets of coefficients differ, and the text below reports β throughout. As shown in Table 3, CFR was significantly and negatively associated with both school relational variables: higher CFR was associated with lower TSR (*β* = −0.332, SE = 0.023, *p* < 0.001; *R*^2^ = 0.121) and lower PRS (*β* = −0.410, SE = 0.022, *p* < 0.001; *R*^2^ = 0.170). In the equation predicting flourishing, both mediators remained significant after mutual adjustment (TSR: *β* = 0.211, SE = 0.023, *p* < 0.001; PRS: *β* = 0.380, SE = 0.023, *p* < 0.001), and the direct association between CFR and flourishing remained significant but was reduced relative to the total effect (*β* = −0.190, SE = 0.021, *p* < 0.001; *R*^2^ = 0.397). Among the covariates, household registration was significantly associated with both mediators (TSR: *β* = −0.110, *p* < 0.001; PRS: *β* = −0.068, *p* = 0.002) and with flourishing in the total-effect model (*β* = −0.082, *p* < 0.001), whereas sex was not significantly associated with any outcome, and only-child status showed only a marginal positive association with flourishing (*β* = 0.036, *p* = 0.057). After CFR and the covariates were controlled, the residual correlation between the two mediators was 0.367 (*p* < 0.001), indicating that the two relational systems overlap yet each retains a substantial unique component.

**Table 3:** Path coefficients of the parallel multiple mediation model (*N* = 1,673).

Outcome/predictor	B	SE	*t*	95% CI	*β*	*R* ^2^
TSR (a₁ path)						
CFR	−0.243	0.017	−14.43	[−0.276, −0.210]	−0.332	0.121
household registration	−0.179	0.038	−4.75	[−0.253, −0.105]	−0.110	
Sex	−0.059	0.048	−1.22	[−0.153, 0.036]	−0.028	
Only child	0.006	0.049	0.13	[−0.090, 0.103]	0.003	
PRS (a₂ path)						
CFR	−0.246	0.013	−18.33	[−0.272, −0.220]	−0.410	0.170
household registration	−0.092	0.030	−3.06	[−0.151, −0.033]	−0.068	
Sex	0.036	0.038	0.94	[−0.039, 0.111]	0.021	
Only child	−0.013	0.039	−0.33	[−0.090, 0.064]	−0.007	
FS (b and c′ paths)						
CFR (c′)	−0.165	0.018	−8.97	[−0.201, −0.129]	−0.190	0.397
TSR (b₁)	0.251	0.027	9.40	[0.198, 0.303]	0.211	
PRS (b₂)	0.549	0.033	16.43	[0.484, 0.615]	0.380	
Sex	0.034	0.047	0.72	[−0.059, 0.127]	0.014	
Only child	0.092	0.048	1.91	[−0.003, 0.186]	0.036	
household registration	−0.063	0.037	−1.68	[−0.136, 0.011]	−0.032	
FS (total effect model, c)						
CFR	−0.361	0.019	−18.70	[−0.399, −0.323]	−0.416	0.178
household registration	−0.158	0.043	−3.66	[−0.242, −0.073]	−0.082	
Sex	0.039	0.055	0.71	[−0.069, 0.147]	0.016	
Only child	0.086	0.056	1.54	[−0.024, 0.197]	0.034	

**Figure 1:**
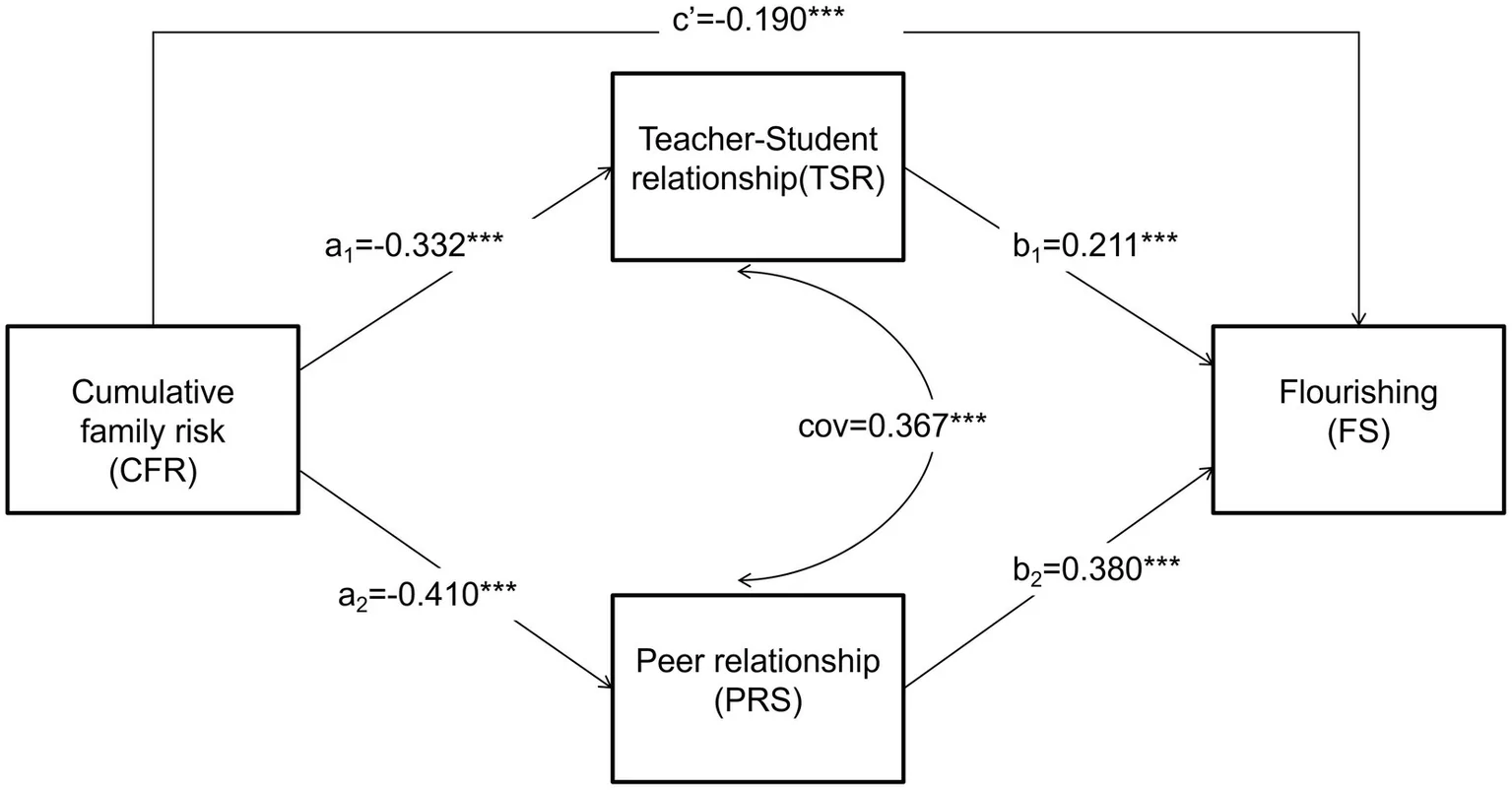
Parallel mediation model. All models are adjusted for sex, only-child status, and household registration. ***p < 0.001.

Table 4 presents the direct and indirect effects. The total effect of CFR on flourishing was −0.416; 54.3% of it was indirect, the total indirect effect through the two school relational channels being −0.226 [BootSE = 0.017, 95% BootCI (−0.261, −0.195)]. Decomposed, the specific indirect effect through peer relationship was −0.156 [BootSE = 0.016, 95% BootCI (−0.188, −0.127)], 37.5% of the total effect, and the specific indirect effect through teacher–student relationship was −0.070 [BootSE = 0.010, 95% BootCI (−0.089, −0.052)], 16.9% of the total effect; the two shares add to 54.4% rather than 54.3% because each is rounded to one decimal. Both were significant, supporting H4 (and, correspondingly, H2 and H3). Expressed within the total indirect effect, the peer pathway accounted for 68.9% and the teacher–student pathway for 31.1%, so that the peer pathway carried 2.22 times the indirect effect of the teacher–student pathway. Unstandardized effects are reported alongside the standardized ones in Table 4.

**Table 4:** Direct, indirect, and total effects of cumulative family risk on flourishing, with the pairwise contrast between the two specific indirect effects (*N* = 1,673).

Effect	B	Boot SE	95% BootCI	*β*	95% BootCI (*β*)	% of total effect
Indirect via TSR (a₁b₁)	−0.061	0.008	[−0.077, −0.046]	−0.070	[−0.089, −0.052]	16.9%
Indirect via PRS (a₂b₂)	−0.135	0.014	[−0.163, −0.110]	−0.156	[−0.188, −0.127]	37.5%
Total indirect effect	−0.196	0.015	[−0.226, −0.169]	−0.226	[−0.261, −0.195]	54.3%
Direct effect (c′)	−0.165	0.018	[−0.201, −0.129]	−0.190	—	45.7%
Total effect (c)	−0.361	0.019	[−0.399, −0.323]	−0.416	—	100%

The pairwise contrast of the two specific indirect effects was significant (a₁b₁ − a₂b₂ = 0.074, BootSE = 0.017, z = 4.26, *p* < 0.001, 95% BootCI [0.041, 0.109]; in completely standardized units, 0.086, 95% BootCI [0.048, 0.125], *p* < 0.001). Because both specific indirect effects are negative, the positive sign of the contrast indicates that the indirect effect carried by peer relationship was larger than that carried by teacher–student relationship; H5 was therefore supported. Expressed in terms of unique explained variance, removing peer relationship from the model reduced the explained variance in flourishing by 9.8 percentage points, whereas removing teacher–student relationship reduced it by 3.2 percentage points.

Because the direct association between CFR and flourishing remained significant after the two mediators were entered, the pattern is one of partial mediation: the remaining 45.7% of the total effect was not accounted for by the two school relational channels assessed here.

### 4.4 Model specification checks

Two specification checks were conducted, both with the three covariates retained and 5,000-resample bias-corrected bootstrap confidence intervals. First, the products of CFR with each mediator were tested. Entered separately, both reached significance [CFR × TSR: B = 0.049, *F*(1, 1,665) = 8.90, *p* = 0.003; CFR × PRS: B = 0.051, *F*(1, 1,665) = 7.32, *p* = 0.007]; entered jointly, neither was individually significant, although their joint contribution was (Wald *χ*^2^(2) = 10.05, *p* = 0.007), the attenuation reflecting the collinearity of the two products (*r* = 0.52). The positive signs indicate that the association between each relational variable and flourishing was slightly stronger at higher levels of family risk, but the increment was small (Δ*R*^2^ = 0.007) and left the sign and significance of every main path unchanged; the additive parallel specification was therefore retained. Second, both serial orderings of the mediators were estimated (Table 5). The specific indirect effects differ across orderings, as they must, because in each chain the path from CFR to the second mediator is adjusted for the first. The total indirect effect (*β* = −0.226) and the direct effect (*β* = −0.190) were identical under both orderings and under the parallel model, because all three specifications share the same final equation and are saturated with respect to the observed covariance matrix. The ordering is therefore not identified by these data, and no claim about temporal precedence is made. Finally, the covariate set was extended as a check: when grade (fourth vs. fifth grade) was added as a fourth covariate, the estimates were unchanged to two decimals (indirect via TSR *β* = −0.071, indirect via PRS *β* = −0.156, total indirect *β* = −0.227, direct *β* = −0.189), and grade was not associated with flourishing (*B* = 0.037, SE = 0.047, *t* = 0.77). All models included sex, only-child status, and household registration as covariates; confidence intervals are bias-corrected bootstrap intervals based on 5,000 resamples. *β* = completely standardized coefficient. In serial specifications the specific indirect effect through the second mediator in the chain uses the CFR → mediator path adjusted for the first mediator, and is therefore not directly comparable with the corresponding parallel-model estimate (Table 4). The total indirect and direct effects are identical across the two orderings and identical to the parallel model.

**Table 5:** Sensitivity analyses of the two serial orderings of the mediators (*N* = 1,673).

Effect	B	95% BootCI	*β*
Teacher-first ordering (CFR → TSR → PRS → FS)			
Indirect via TSR (a₁b₁)	−0.061	[−0.075, −0.048]	−0.070
Indirect via PRS (a₂b₂, adjusted for TSR)	−0.089	[−0.109, −0.073]	−0.103
Serial path (CFR → TSR → PRS → FS)	−0.046	[−0.055, −0.038]	−0.053
Peer-first ordering (CFR → PRS → TSR → FS)			
Indirect via PRS (a₂b₂)	−0.135	[−0.159, −0.114]	−0.156
Indirect via TSR (a₁b₁, adjusted for PRS)	−0.028	[−0.037, −0.019]	−0.032
Serial path (CFR → PRS → TSR → FS)	−0.033	[−0.043, −0.023]	−0.038
Total indirect effect (both orderings)	−0.196	[−0.221, −0.173]	−0.226
Direct effect (c′)	−0.165	[−0.197, −0.133]	−0.190

## 5 Discussion

### 5.1 Impact of CFR on children’s flourishing

The findings show that CFR is negatively associated with primary school children’s sense of flourishing. Furthermore, this negative association shows a cumulative pattern: flourishing is lower in a stepwise, approximately linear fashion as the total number of risk factors increases. This is consistent with the view that cumulative risk is related to lower flourishing through a progressive depletion of psychological resources. The CFR perspective draws attention to the covariation between a dysfunctional family environment and a child’s psychological development, rather than to individual family adversities.

No accelerating or threshold pattern was detected in the present sample. For prevention, this implies that the count of co-occurring risks matters at every level, and that deferring action until a presumed threshold is reached would leave accumulated harm unaddressed. This is also consistent with the additive construction of the cumulative risk index itself (Evans et al., 2013).

First, cumulative risk is more than the simple sum of individual factors; the accumulation itself carries an added toll. When the total number of risk factors exceeds a child’s maximum psychological defense capacity, the continuous deprivation of basic psychological needs has been found to accompany severe systemic damage (Liu et al., 2026). Second, different risk-factor configurations often lead to similar adverse outcomes. Therefore, assessing the cumulative total risk is more effective at predicting the extent of the loss of a child’s sense of flourishing than tracking individual risk factors (Liu et al., 2025). Higher levels of CFR were accompanied by poorer interpersonal adaptation in the school environment, consistent with the notion that dysfunction in the family microsystem crosses system boundaries. Children exposed to multiple risks reported greater emotional exhaustion, which may reflect the long-term excessive emotional burden placed on their psychological defense systems (Zhao and Wang, 2025; Yuan et al., 2021).

A poor family atmosphere often accompanies a weak parental relationship. Poor family economic conditions are linked to parents’ relatively low educational levels, and an incomplete family structure is associated with a deficient parenting style. Overall, various family risk factors present a symbiotic rather than an isolated situation (Pan et al., 2025; Zhao and Wang, 2025). As the cumulative total number of risk factors rises, psychological resources are progressively depleted (Yang et al., 2023; Zeng et al., 2025). Sustained exposure of this kind is accompanied by lower self-esteem and by fewer opportunities to obtain social support, two proximal correlates of lower flourishing.

### 5.2 The mediating role of teacher–student and peer relationships

The findings reveal that both teacher–student as well as peer relationships play independent mediating roles in the relationship between CFR and the primary school children’s flourishing. Family dysfunction, the most pervasive microsystem during an individual’s development, can interfere with children’s interpersonal adaptation to the school microsystem.

The teacher–student relationship, as a vertical support path, is related to systemic pressure from the family. According to the family stress model, deteriorating parent–child interaction, family financial difficulties, and long-term stress have been found to generate negative spillover effects, which co-occur with social withdrawal or adaptation difficulties in children when they encounter a non-relative authoritative adult like a teacher (Chen and Wu, 2025). Therefore, a supportive teacher–student relationship is associated with a higher sense of self-worth and positively predicts students’ subjective well-being (Zhang et al., 2025). Meta-analytic evidence corroborates that affective teacher–student relationships are reliably associated with students’ school engagement (Roorda et al., 2011). However, a decline in its quality implies the loss of the necessary psychological buffer and a lower standing on the “positive psychological function” dimension of flourishing (Hernández et al., 2021). Even in economically disadvantaged families, where children receive timely care in a positive atmosphere and their psychological needs are met, children develop secure attachment and show better development (Wang et al., 2022). Therefore, focusing on one type of family risk may not provide a comprehensive view of the outcomes related to the formation of children’s interpersonal relationships. This points to the value of the CFR perspective for interpreting this issue.

Moreover, peer relationships, as a horizontal dimension of social resources, decline as family risk factors accumulate. Harmonious parent–child relationships can enhance children’s communication skills, positive emotions, and interpersonal abilities, which are essential qualities for forming high-quality friendships (Yang et al., 2025). Negative parenting styles or parental conflict can disrupt children’s basic psychological needs, causing them to exhibit either hostile or withdrawn behaviors in peer interactions (He et al., 2023; Guo et al., 2020). High-quality friendships are associated with fewer depressive symptoms in children. Strong peer support can reduce feelings of loneliness and psychological discomfort, and promote positive emotional development (Yang et al., 2025). The small positive interactions suggest that these protective associations were somewhat stronger among children facing higher cumulative family risk, consistent with school relationships functioning as compensatory resources precisely where family resources are most depleted.

When children report a less stable peer support network in the context of family structure, economic pressure, or digital neglect, their sense of belonging is compromised, as are the social functions required for flourishing (Bao et al., 2025). Taken together, the accumulation of family risks is associated with lower flourishing, and this association is accounted for in part by the concurrent erosion of children’s compensatory social resources at school.

### 5.3 Relative magnitude of the two relational pathways

The central finding of this study is not that both relational systems carry part of the association between family cumulative risk and flourishing, but that they do so to clearly different degrees. Both pathways contributed independently, consistent with H4, yet the peer pathway carried the larger share of the total association and the teacher–student pathway a smaller share. A formal pairwise contrast of the two specific indirect effects was consistent with H5. The same asymmetry appeared in the unique explained variance: removing peer relationship from the model reduced the explained variance in flourishing more than removing teacher–student relationship did. Taken together, the two relational channels accounted for a substantial share of the total association between CFR and flourishing, with the peer channel carrying the larger part of that share.

This asymmetry is consistent with theoretical expectations rather than anomalous. By Grades 4 to 5, the peer group has become a consolidated social context in which children spend the largest share of their school day and from which they draw most of their daily social validation (Rubin et al., 2006). At the same time, the mechanism through which teachers influence a child’s standing among classmates is concentrated in negative teacher behavior and is considerably weaker for positive relationship quality (Endedijk et al., 2022; Hendrickx et al., 2017); because the present study measured the positive quality of both relationships, the teacher-to-peer channel was assessed at its weaker end. Finally, longitudinal evidence gathered in the same grade range indicates that peer acceptance and rejection predict later teacher–student relationship (De Laet et al., 2014; Deng et al., 2025), which is compatible with the peer channel carrying the larger share of the transmission. This pattern is also consistent with the view that by late childhood peer relationships are already highly developed and exert independent influence on adjustment.

The asymmetry should not be read as evidence that teacher–student relationships are dispensable. Their contribution persisted after peer relationship was controlled, and meta-analytic work indicates medium-to-large associations between affective teacher–student relationships and students’ school engagement (Roorda et al., 2011). Teachers also occupy a distinctive structural position: they are frequently the first adults to detect that a child is carrying family adversity, and their public behavior toward a child shapes how classmates treat that child (Hendrickx et al., 2017). Our findings therefore address the prioritization of intervention resources, not the exclusion of either channel.

Two caveats are necessary. First, because the data are cross-sectional, the model estimates a theory-driven decomposition of covariance and does not establish that family risk causes changes in school relationships which in turn cause changes in flourishing; cross-sectional estimates of longitudinal mediation can be biased even when mediation is complete (Maxwell and Cole, 2007). Second, the ordering of the two mediators is not identified by the present data, and no claim about their temporal precedence is made. Finally, a substantial portion of the total association remained direct, indicating that family cumulative risk reaches children’s flourishing through additional channels not assessed here, such as emotion regulation, academic self-concept, and material resources.

### 5.4 Theoretical implications and practical interventions

Theoretically, this study used an empirical model to elucidate how family risk factors systematically erode children’s positive developmental potential. First, it shifts focus from traditional maladjustment, such as depression, to flourishing (Rule et al., 2024). This shift aligns with positive psychology’s emphasis on individual functioning. Second, by specifying the two school relational systems as parallel mediators and contrasting their magnitudes, the study reformulates the question of cross-system interpersonal penetration. Rather than asking which relational resource is depleted first—a question that cross-sectional data cannot answer—it asks how much of the family-risk transmission each resource carries, a question that such data can answer. Third, the observed asymmetry refines the account of cross-system penetration offered by the family stress model: family adversity does not erode school relational resources uniformly, and the horizontal peer channel appears to be the more heavily loaded conduit at this age.

Regarding practical interventions, the results point to the value of identifying and classifying support needs. Educators should focus on CFR rather than on individual factors. By identifying the association between total risk factors and children’s flourishing, they can more precisely identify children who are under pressure and whose psychological states are most vulnerable. Because the peer pathway carries the larger share of the indirect effect, and because it accounts for a larger share of unique variance in flourishing, intervention resources should be directed first toward restoring and consolidating peer relationships for children exposed to multiple family risks—through structured cooperative learning, peer-pairing arrangements, and systematic antibullying practices. Two further implications follow. Teacher–student relationship work should be retained rather than displaced, given its independent contribution and teachers’ structural position as the adults best placed to detect risk early. And because teachers most strongly shape a child’s standing among classmates through their negative behavior, a low-cost and well-supported recommendation is that teachers avoid expressing criticism or dislike toward an at-risk child in public, correcting behavior privately instead.

### 5.5 Limitations and future research directions

This study investigated the synergistic influence of family and school microsystems on children’s psychological development, yet several limitations remain. First, cross-sectional data were utilized in this research. Although we modeled the two school relational systems as parallel mediators, the cross-sectional design cannot establish definitive causal sequences among variables, and the estimates obtained may diverge from the longitudinal parameters they are intended to approximate (Maxwell and Cole, 2007). Consequently, the present study refrains from any claim about temporal precedence. These alternative specifications are reported for completeness. Subsequent multi-wave longitudinal designs (e.g., Random Intercept Cross-Lagged Panel Model) are recommended to test both the direction of influence between the two relational systems and the temporal stability of the pathways reported here.

Second, the data collection was entirely based on students’ self-assessment, which may be affected by common method bias or social approval. Subsequent research should introduce multi-source evaluation methods, including teacher assessment, peer nomination, or parent reports to enhance the authenticity and comprehensiveness of the research conclusions. Third, the sample was solely from the northwest region of China, which may limit the result’s generalizability to different cultural or socio-economic background groups. Future research should assess the universality of the CFR on children’s flourishing in a broader area. Fourth, the cumulative risk index is by construction an unweighted count of dichotomized adversities; it cannot speak to the possibly differential weighting of specific risk factors, and risk intensity within each factor is not retained (Evans et al., 2013). Fifth, this study mainly focused on the depletion path of school interpersonal support. Future studies can explore individual internal protective factors, such as psychological resilience and self-efficacy, to further analyze how individuals balance external risks and internal resources to cope with environmental pressures to maintain flourishing. Sixth, Class and school identifiers were not retained in the analysis file, so the intraclass correlation could not be estimated and cluster-robust or multilevel standard errors could not be computed. Dependence among observations within the same class, if present, would leave the point estimates unchanged but could reduce the precision of the significance tests reported here; school-based relational mediators should therefore be re-examined with the nesting modelled explicitly.

## 6 Conclusion

This study shows that the accumulation of family risks is associated with lower levels of positive development among primary school children. This negative association increases in an approximately linear fashion as the total number of risk factors increases, with no evidence of an accelerating or threshold effect within the observed risk range. Difficulties prevalent in the family environment are echoed in the school environment, where they co-occur with poorer interpersonal relationships and lower flourishing among children. Teacher–student and peer relationships function as two parallel channels linking the family environment and children’s growth, and together they carry a substantial share of the total association between cumulative family risk and flourishing. The two channels are unequal in capacity: the peer pathway carried the larger share of the transmission, a difference that is theoretically expected at this age. A substantial portion of the total association remained direct, indicating that family cumulative risk reaches children’s flourishing through channels beyond the two assessed here. Therefore, educators should prioritize children facing several family pressures, and—where resources are constrained—should give precedence to restoring peer relationships, while continuing to sustain teacher–student relationships as an independent and non-substitutable channel for promoting children’s psychological flourishing.

## Data Availability

The raw data supporting the conclusions of this article will be made available by the authors, without undue reservation.
